# SARS‐CoV‐2 Omicron variant: Immune escape and vaccine development

**DOI:** 10.1002/mco2.126

**Published:** 2022-03-16

**Authors:** Danyi Ao, Tianxia Lan, Xuemei He, Jian Liu, Li Chen, Daniel T. Baptista‐Hon, Kang Zhang, Xiawei Wei

**Affiliations:** ^1^ Laboratory of Aging Research and Cancer Drug Target State Key Laboratory of Biotherapy and Cancer Center National Clinical Research Center for Geriatrics West China Hospital Sichuan University Chengdu Sichuan China; ^2^ Center for Biomedicine and Innovations Faculty of Medicine Macau University of Science and Technology Macau China

**Keywords:** immune escape, Omicron variant, spike, vaccine development

## Abstract

New genetic variants of severe acute respiratory syndrome coronavirus 2 (SARS‐CoV‐2) constantly emerge through unmitigated spread of the virus in the ongoing Coronavirus disease 2019 pandemic. Omicron (B.1.1.529), the latest variant of concern (VOC), has so far shown exceptional spread and infectivity and has established itself as the dominant variant in recent months. The SARS‐CoV‐2 spike glycoprotein is a key component for the recognition and binding to host cell angiotensin‐converting enzyme 2 receptors. The Omicron variant harbors a cluster of substitutions/deletions/insertions, and more than 30 mutations are located in spike. Some noticeable mutations, including K417N, T478K, N501Y, and P681H, are shared with the previous VOCs Alpha, Beta, Gamma, or Delta variants and have been proven to be associated with higher transmissibility, viral infectivity, and immune evasion potential. Studies have revealed that the Omicron variant is partially resistant to the neutralizing activity of therapeutic antibodies and convalescent sera, which poses significant challenges for the clinical effectiveness of the current vaccines and therapeutic antibodies. We provide a comprehensive analysis and summary of the epidemiology and immune escape mechanisms of the Omicron variant. We also suggest some therapeutic strategies against the Omicron variant. This review, therefore, aims to provide information for further research efforts to prevent and contain the impact of new VOCs during the ongoing pandemic.

## Graphical abstract

Omicron (B.1.1.529), the latest variant of concern, is partially resistant to the neutralizing activity of therapeutic antibodies and convalescent sera, which poses significant challenges for the clinical effectiveness of the current vaccines and therapeutic antibodies. We provide a comprehensive analysis and summary of the epidemiology and immune escape mechanisms of the Omicron variant. We also suggest some therapeutic strategies against the Omicron variant.

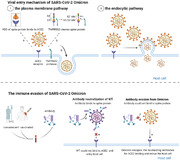

## 1 INTRODUCTION

The global outbreak of Coronavirus disease 2019 (COVID‐19) has been declared a pandemic since March 2020. Despite an unprecedented global effort to develop vaccines and treatment strategies, the pandemic is showing little signs of diminution, driven mostly by the emergence of new variants. COVID‐19 is caused by an RNA virus, the severe acute respiratory syndrome coronavirus 2 (SARS‐CoV‐2). Consistent with most RNA viruses, the RNA‐dependent RNA polymerase (RdRp) of SARS‐CoV‐2 incorporates mismatches during the replication of the viral genome, resulting in relative instability of the SARS‐CoV‐2 genome. This instability, in combination with a selection pressure, drives the emergence of genetic diversity and evolution of SARS‐CoV‐2.^1^, ^2^ The end result of this genetic diversification and evolution is the emergence of variants.

To prioritize global monitoring and research on SARS‐CoV‐2, the World Health Organization (WHO) classified SARS‐CoV‐2 variants into three categories: variants of concern (VOCs), variants of interest (VOIs), and variants under monitoring. At the time this review was written, there were five VOCs, including Alpha (B.1.1.7), Beta (B.1.351), Gamma (P.1), Delta (B.1.617.2), and Omicron (B.1.1.529).^3^ The naming of these variants follows a chronological order.^4^ The Alpha, Beta, Gamma, and Delta VOCs have shown progressive changes in their virology, particularly in regards to their transmissibility and disease severity. Therefore, the emergence of the Omicron variant has brought huge concerns about its potential threat to public health and economy. Initial genetic sequence analyses of the Omicron variant revealed more than 60 alterations in the genome, which make it the most mutated VOC so far.^5^ Many of these alterations are concentrated in the spike protein region, which in theory may substantially impair the efficacy of the current COVID‐19 vaccines. Initial reports and information from South Africa also suggest a substantially higher transmissibility, raising great concerns about the prevention and control of this wave of COVID‐19 epidemic.

At the time this review was written, more than 430 million people have been diagnosed with COVID‐19 globally, resulting in 5.9 million deaths.^6^ In particular, the Omicron has resulted in a surge in infections in many countries and regions since its identification. Especially, the confirmed COVID‐19 cases in the United States exceeded one million in a single day in early January 2022.^7^ This sharp increase is consistent with the outbreak of the Omicron variant in the United States.

## 2 EPIDEMIOLOGY AND FEATURES OF THE OMICRON VARIANT

### 2.1 Epidemiology of the Omicron variant

**FIGURE 1:**
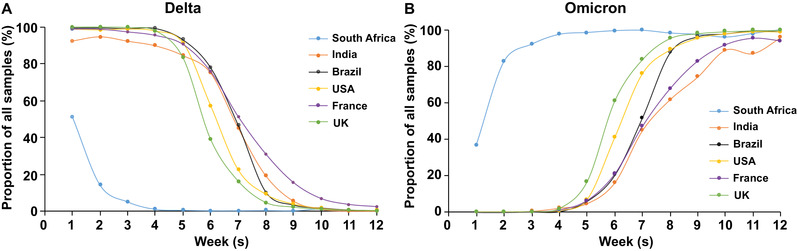
The estimated proportion curve of severe acute respiratory syndrome coronavirus 2 (SARS‐CoV‐2) Delta variant and Omicron variant in total samples. The proportion curve of Delta (A) and Omicron (B) cases were shown weekly from November 8, 2021, to January 24, 2022, in six countries (South Africa, India, Brazil, the United States, France, and the United Kingdom). Proportion is relative to all samples collected. Data were obtained from GISAID and accessed on February 12, 2022.^9^

The earliest Omicron infection discovery could trace back to November 9, 2021, in South Africa. The first complete Omicron sequence was obtained from a sample collected on November 11, 2021, in Botswana.^8^ We downloaded available data from covSPECTRUM regarding confirmed cases of different VOCs in different countries. Figure 1 shows the proportion of confirmed cases attributed to the Delta (Figure 1A) and Omicron (Figure 1B) variants. Our analysis revealed that the Delta variant was the dominant strain in the world prior to the emergence of the Omicron variant. Since the identification of Omicron, it has spread rapidly in South Africa. By November 13, 2021, 80% of the sequence results of 266 samples were attributed to the Omicron variant (Figure 1B).^9^ Subsequently, the daily number of COVID‐19 cases in South Africa increased sharply, from 305 (November 11, 2021) to a peak of 37,875 (December 13, 2021).^10^

Some European countries also reported cases of Omicron simultaneously, which indicated that this variant might have spread widely in many countries before it was discovered in South Africa.^11^ The estimated proportion curve of the Omicron variant in total samples in South Africa, India, Brazil, the United States, France, and the United Kingdom are shown in Figure 1B. We observed that the Omicron variant spread quickly in the United Kingdom in December. The proportion of Omicron in all sequenced samples has increased from ∼5% in early December to ∼95% at the end of December 2021.^12^ Subsequently, the Omicron almost completely replaced Delta and led to a new wave of the pandemic in the United Kingdom. According to Global Influenza Surveillance and Response System (GISAID), a similar situation of Omicron replacing the previous existing variants had also occurred in some other countries, such as India, the United States, Brazil, and France (Figure 1B). An epidemiological study from India illustrated that Omicron had caused a third wave of infection in the vast majority of India, which exerted a higher transmission rate and infectivity than the previous waves of COVID‐19 outbreaks.^13^ More importantly, more than 98.11% of global sequences were identified as Omicron on February 2022 according to GISAID, indicating the dominant prevalence of Omicron in the world.^14^ As of February 28, 2022, the Omicron has spread in 157 countries and has resulted in a sharp increase of COVID‐19 cases in many countries and regions.^15^

### 2.2 Clinical features of the Omicron variant induced COVID‐19

The Omicron variant has a 13‐fold increase in infectivity, around 2.8 times more infectious than the Delta variant.^16^ The basic reproductive number (R_0_), the average number of additional cases generated by a single infected individual, for the Omicron variant is also increased. The original strain of SARS‐CoV‐2 has an R_0_ of 2–3, and the Delta variant has an R_0_ of 5–8, while Omicron's R_0_ is estimated as high as 10.^17^, ^18^, ^19^ Moreover, two studies from Denmark and South Africa reported that the effective repopulation number of Omicron was 3.19 times (95% confidence interval (CI): 2.82∼3.61) and 4.2 times (95% CI: 2.1∼9.1) higher than that of Delta.^20^, ^21^ The early doubling time for the Omicron variant (1.2 days) is also faster than the other VOCs (Beta: 1.7 days, Delta: 1.5 days).^8^ This is consistent with a shorter incubation period for the Omicron variant (3 days), compared with the wild‐type (WT; 5 days) and Delta (4 days) variants.^22^, ^23^ Taken together, these studies demonstrate that the Omicron variant has greater transmissibility than other VOCs.

One important observation in the early outbreak of the Omicron variant in South Africa was the relatively milder symptoms. A study in Gauteng, South Africa, compared the hospitalization rate, severity of patients, and mortality rate during the Beta, Delta, and Omicron waves.^24^ While there were more confirmed cases attributed to the Omicron variant, the proportion of cases requiring hospitalization was 4.9%. This is substantially lower than that recorded during the Beta and Delta outbreaks, with hospitalization rates between 13.7% and 18.9%. However, hospitalization rates for Omicron were higher in the less than 20 years of age subgroup, suggesting that Omicron infection may be more detrimental in the younger population. However, this could be due to a lower vaccination rate in this group. The disease severity of hospital‐admitted patients with Omicron was also lower. A cross‐sectional comparison of the deaths caused by the three different variants showed that the mortality rate of Omicron was the lowest in all age groups. In general, fewer patients required oxygen therapy. A lower proportion of patients required intensive care unit (ICU) treatment, and the median length of stay in the hospital was also shorter. These observations were consistent with a clinical comparative analysis of patients infected with Delta and Omicron variants where emergency department visits, hospitalization, ICU admission, and mechanical ventilation were lower in those infected with the Omicron variant.^25^ Another study with children under 5 years of age, using the same outcomes to assess the severity of Omicron, revealed similar results.^26^ Furthermore, the patients suffer from the commonest symptoms including runny nose, headache, and fatigue.^27^, ^28^ Taken together, these results indicated that the Omicron variant resulted in mild symptoms and a lower rate of hospitalization and mortality, compared to the Delta variant.

Pneumonia is the predominant symptom of COVID‐19 patients infected with other VOCs. However, analysis of clinical and epidemiological characteristics showed that only a small proportion of Omicron infections displayed lung infiltrations consistent with pneumonia on chest image, and the majority presented with symptoms more akin to an upper respiratory tract infection.^28^, ^29^ In vivo studies with hamsters also revealed that the Omicron variant was less likely to infect the lungs when compared with the Delta variant.^30^ In addition, in vitro studies also showed that the Omicron variant replicated faster than any other SARS‐CoV‐2 variants (e.g., 100‐fold more rapidly than the Delta variant) in human primary nasal epithelial cells.^31^, ^32^ This extraordinary replication rate of the Omicron variant in nasal epithelial cells may result in a higher viral load in the upper respiratory tract, which would lead to the acceleration of the transmission rate. Interestingly, the replication rate of the Omicron variant was shown to be lower in human alveolar cells, compared to that of the Delta variant.^33^ The Omicron variant also showed impaired S1/S2 cleavage and decreased efficiency of utilizing host transmembrane protease serine type 2 protein, required for viral entry into host cells.^34^, ^35^ The Omicron variant also induced lower activation of nuclear factor kappa B (NF‐κB) pathway than the Delta variant, which may partly explain the milder symptoms associated with Omicron infections.^36^ These unique features of the Omicron variant can account for the lesser inflammatory response and impaired fusion with host lung cells, thus resulting in a unique epidemiology of high transmissibility but mild disease.^37^

### 2.3 Mutation characteristics

Phylogenetic analysis of SARS‐CoV‐2 genetic sequences reveals that the Omicron variant has two subtypes, BA.1 and BA.2.^38^ BA.1 is responsible for the initial Omicron outbreak, and at the time of writing, is the predominant subtype worldwide. Whole‐genome analysis of the BA.1 and BA.2 subtypes found more than 60 non‐synonymous mutations, including base substitutions, deletions, and insertions.^39^ A substantial number of these mutations are concentrated in the spike protein region. Specifically, BA.1 and BA.2 display 20 identical spike mutations, which are G339D, S373P, S375F, K417N, N440K, S477N, T478K, E484A, Q493R, Q498R, N501Y, Y505H, D614G, H655Y, N679K, P681H, N764K, D796Y, Q954H, and N969K. Additional mutations are found in the BA.1 spike protein, including 10 substitutions (A67V, T95I, Y145D, L212I, S371L, G446S, G496S, T547K, N856K, and L981F), three deletions (H69‐/V70‐, G142‐/V143‐/Y144‐, and N211‐) and a three amino‐acid insertion at position 214.

Other structural proteins of BA.1 also display mutations. These include T9I in the envelope (E) protein, D3G, Q19E, and A63T in the membrane (M) protein, and a number of deletions (E31‐, R32‐, S33‐) and substitutions (P13L, R203K, and G204R) in the nucleocapsid (N) protein. Non‐structural proteins also display mutations. For instance, ORF1a shows five substitutions (K856R, L2084I, A2170T, T3255I, and P3395H) and four deletions (S2083‐, L3674‐, S3675‐, and G3676‐). ORF1b has two substitutions (P314L and I1566V). ORF9b has substitution (P10S) and three deletions (E27‐, N28‐, and A29‐).

**FIGURE 2:**
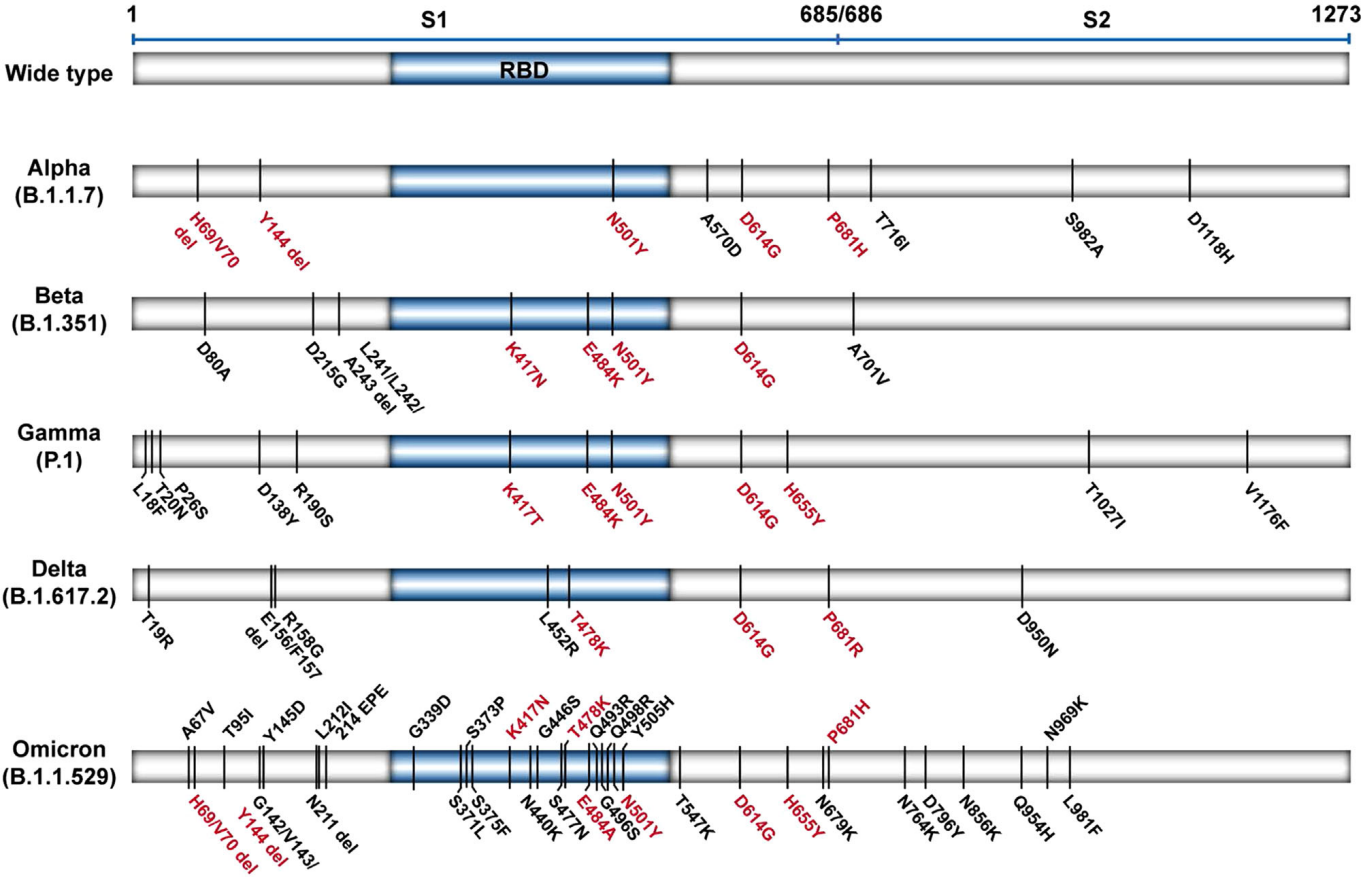
The schematic representation of the mutations in the spike protein of five SARS‐CoV‐2 variants of concern (VOCs). Mutation data were from CoVariants.^5^ The same and similar mutations (red word) among those VOCs are highlighted. Omicron variant mutations are based on 21K or BA.1. RBD, receptor‐binding domain

It is clear that the Omicron variant has a large number of mutations, compared to other variants (Figure 2). Notably, many of these mutations are within the sequences encoding receptor‐binding domain (RBD), raising the possibility of (1) altered binding affinity to host angiotensin‐converting enzyme 2 (ACE2) and (2) altered affinity to therapeutic antibodies. The T478K substitution, found in Delta and Omicron variants, is located within the RBD of the spike protein.^40^ This non‐conservative substitution (uncharged amino acid to a positively charged amino acid) may impact any electrostatic interactions between the spike protein and ACE2.^40^, ^41^ Although the Omicron‐specific RBD substitutions (K417N and E484A) reduced binding of the spike protein to ACE2, other mutations that increased the affinity for ACE2 could compensate for such effects.^42^, ^43^ One example is the N501Y mutation, shared by Alpha, Beta, and Gamma variants, which enhances the binding of spike to ACE2.^44^, ^45^ Yeast surface display technology revealed that spike proteins harboring the E484K/N501Y double mutations could induce stronger affinity than the N501Y alone.^46^ Interestingly, the presence of the K417N mutation did not affect spike‐ACE2 binding affinity but produced positive cooperativity with E484K/N501Y.^46^ Thus, the presence of the three mutations N501Y/E484A/K417N may increase the binding affinity of spike protein to ACE2 and raise the possibility that this triple mutation may enhance the transmissibility of the Omicron variant.

The Omicron variant also harbors a unique cluster of mutations in the RBD. The N440K and Y505H substitutions, found only in the Omicron variant, are associated with increased infectivity.^47^, ^48^ The S477N substitution was also reported to enhance the binding between spike protein and ACE2, leading to a slight increase in infectivity, compared with N501Y.^48^, ^49^ Structural modeling and comparisons of the binding interface between the spike protein and ACE2 in Omicron and Delta variants suggest the three mutations (Q493R, G496S, and Q498R) found only in the Omicron variant formed additional interactions with ACE2. There are new salt bridges formed by the Q493R and Q498R substitutions.^42^ The G496S substitution formed a new hydrogen bond not presented in the Delta variant.^42^ Therefore, it would appear that these three mutations in the Omicron variant increased the number of interactions with ACE2. Furthermore, consistent with the observations highlighted above, the Q498R substitution may act synergistically with the existing N501Y substitution to increase the affinity of the spike protein to ACE2.^46^ The impact of other novel substitutions in the Omicron RBD (e.g., G339D, S371L, S373P, and S375F) are suggested to have relatively milder impacts on the binding affinity to ACE2.^16^ However, further studies are required to fully elucidate their functions. Mutations in other SARS‐CoV‐2 proteins can also lead to increased infectivity of the Omicron variant. For example, the H69‐/V70‐ deletions can induce S gene targeting failure, leading to increased levels of cleaved S2 protein and higher infectivity of the virus.^50^

Not all mutations in the spike protein could lead to increased binding affinity to ACE2. The D614G mutation, present in all VOCs, is associated with higher viral load in the upper respiratory tract of younger patients.^51^ Previous studies illustrated that D614G reduces the binding affinity to ACE2 but enhances the protease cleavage of S1/S2, leading to higher transmissibility.^52^, ^53^ Another example is the P681H mutation located in the furin protease cleavage site. This non‐conservative substitution (non‐polar amino acid to a positively charged polar amino acid) may result in more efficient spike protein cleavage.^54^ The H655Y substitution is also found in the furin cleavage site. While this mutation causes only a modest increase in the binding affinity to ACE2 (1.2‐fold), these mutations may account for the enhanced spread of the Omicron variant, through enhanced proteolytic cleavage.^55^ Additional mutations found in the Omicron variant (e.g., A67V, T95I, G142‐/V143‐/Y144‐/Y145D, and N211‐/L212I) are associated with infectious capacity, but their precise role remains to be elucidated.^56^

In summary, these mutations alter the conformation of the RBD, making Omicron easier to bind to ACE2 than SARS‐CoV‐2 WT, which finally makes the significantly higher transmissibility and infectivity.^42^, ^43^

## 3 IMMUNE EVASION OF SARS‐COV‐2 OMICRON

### 3.1 Mechanisms of viral immune evasion

The evasion of the immune system by viruses has been recognized for some time.^57^, ^58^ In general, viruses utilize three main immune evasion strategies^59^, ^60^, ^61^, ^62^: (1) impairment of the humoral immune response; (2) Interruption of the cellular immune response; (3) impairment of immune effectors such as cytokines and apoptosis‐related proteins. The humoral immune response mediates the production of antibodies against the virus. Escape from this humoral immune response was the first identified mechanism of viral immune evasion.^58^ The relative instability of viral genomes (particularly for RNA viruses such as SARS‐CoV‐2) leads to antigenic variability, which underlies this mechanism of escape.^63^ The instability of the SARS‐CoV‐2 genome is mainly caused by the relatively low fidelity of the RdRp, leading to a random mutation rate higher than that for DNA viruses.^64^ These mutations may substantially impair the binding affinity of antibodies as we have outlined above.^65^ On the other hand, viral immune evasion is also associated with the impairment of the non‐specific effector of humoral immunity, the complement system.^66^ Some gene sequences in the viral genome are homologs encoding the complement regulatory proteins that could inhibit the activation of the complement system. For example, it has been demonstrated that the genome of herpesvirus saimiri contains the homolog of the cellular membrane glycoprotein CD59. This protein could target membrane attack complex, an important effector of the complement system.^67^

The cellular immune response plays a crucial role in the clearance of viruses. In particular, CD8^+^ T cells, CD4^+^ T cells, and natural killer (NK) cells participate in the elimination of virus‐infected cells.^68^ Nevertheless, a series of strategies have been developed by viruses to evade the cell‐mediated immune response.^69^ First, it has been revealed that viruses can hamper the process of proteasomal degradation. For instance, Epstein–Barr nuclear antigen 1 expressed by Epstein–Barr virus was reported to drive the host cell to express a cis‐acting inhibitor of ubiquitin‐proteasome proteolysis.^70^ Second, viruses are able to block the presentation of antigens through virus‐induced degradation of major histocompatibility complex (MHC) class I, thereby affecting the activation of CD8^+^ T cells and CD4^+^ T cells.^71^ Third, viruses can evade the NK cell‐mediated killing by expressing proteins that interact with killer cell inhibitory receptors. For instance, it has been demonstrated that human cytomegalovirus express class I MHC homolog to evade the NK cell‐mediated killing of the host cells.^72^

Specifically for the Omicron variant, mutations within the spike protein affect the binding affinity between the virus and ACE2. Aside from contributing to a higher infectivity, this will also enhance competition for RBD binding sites since both neutralizing antibodies and ACE2 bind to this part of the spike protein. In addition, the mutations may also change the epitope for neutralizing antibodies.

In terms of evasion of cell‐mediated immunity, available information suggests that this immunity induced by vaccines or previous infections remains effective against the Omicron variant.^73^, ^74^, ^75^ However, some mutations, particularly in the ORFs may play a role in immune evasion of the Omicron variant. For example, deletions in ORF1a (L3674, S3675, and G3676) may enhance immune evasion by suppressing viral autophagy.^76^, ^77^ Additionally, deletions in ORF9b (E27, N28, and A29) were reported to suppress the host's innate immune response through regulating the production of interferons mediated by the mitochondrial outer membrane protein (TOM70) and the NF‐κB essential modulator (NEMO).^78^, ^79^ Notably, the deletion of 30 amino acids in the N‐terminal domain of ORF9b inhibited the association with NEMO.^80^ For the remainder of this review, we will focus on the evasion of humoral and antibody‐mediated immune responses by the Omicron variant.

### 3.2 Influence of mutations of the Omicron variant on the immune evasion

Monoclonal antibody (mAb) treatment and vaccines that are currently approved or under development target the spike protein ACE2 interaction.^81^, ^82^ However, the considerable number of mutations in the spike protein may affect the binding of antibodies. Serial mutagenesis of two positions on the Omicron variant spike protein identified seven mutations that enhanced immune escape.^83^ Three of these mutations are in position 477 (S477N, S477G, and S477R) and four in position 484 (E484A, E484D, E484G, and E484K). Interestingly, S477N and E484A are found in the Omicron variant. S477N and E484A showed high resistance to multiple mAbs in neutralization assays. Using four different sera from recovered subjects, E484A allowed the Omicron variant to escape the neutralization of all four sera and S477N allowed the escape of two out of four sera.^83^ Mutations in the adjacent T478 also showed resistance to some mAbs and sera.^83^ Mutations to E484 can also enhance the antibody escape capabilities conferred by existing or known mutations in SARS‐CoV‐2. For instance, the K417N and N501Y mutations were known to confer protection against a number of mAbs. However, in the presence of E484K, the protection range could be extended.^84^ Although E484K is not known to exist in the Omicron variant, the analogous E484A can be reasonably speculated to perform a similar role.

Other mutations that confer the ability to escape antibody neutralizing activities in the Omicron variant spike protein include Q493R and G446S, which affected the neutralizing activities of mAbs as well as polyclonal sera, while S371L affected four RBD classes of mAbs.^85^ In addition, SARS‐CoV‐2 variants (e.g., Omicron) with N440K mutation are also more likely to escape antibody neutralization activities.^86^ Specific sites in the spike protein are recurrent deletion regions, which are hotspots for deletion mutations. The deletions, Δ141–144, Δ144/145 and Δ144/145, all allowed escape from the neutralization activity of antibodies, whereas the Δ69–70 deletions alone had only allowed partial escape.^87^

### 3.3 Evasion by Omicron from immunity induced by vaccines

Early studies following the emergence of the Omicron variant showed a marked reduction in the efficacy of the major approved vaccines, such as BNT162b2 (Pfizer‐BioNTech), mRNA‐1273 (Moderna), Ad26.COV2.S (Johnson‐Johnson), and ChAdOx1 nCoV‐19 (Astra Zeneca). There was little inhibition of the Omicron variant by the sera from subjects fully vaccinated with ChAdOx1 nCoV‐19 or BNT162b2.^88^ Also, significant drops in the ID50, a measure of neutralizing activity, were observed in subjects who were vaccinated with the major vaccines. The neutralization activity against the Omicron variant by the BNT162b2 or mRNA‐1273 vaccines was reduced by over 21‐fold and 8.6‐fold, respectively, when compared with their neutralizing activity against the Delta variant. We outlined above that the S371L, N440K, G446S, and Q493R substitutions participated in the escape of antibody neutralization of the Omicron variant.^85^ In a population study of BNT162b2 vaccinated Africans, the Omicron variant shows a 22‐fold increase of escape from neutralization when compared with the ancestral SARS‐CoV‐2 strain.^89^

Clinical data regarding the effectiveness of vaccines against the Omicron variant is also available. An mRNA vaccine ARCoV, currently in the multi‐regional Phase 3 (NCT04847102), encodes the RBD of the WT spike protein. Neutralizing activity against Omicron variants was assessed using serum samples from participants in Phase 1 clinical trial of the ARCoV vaccine. The results indicated that neutralizing activity against Omicron was detectable in most samples, but the antibody titer was approximately 47‐fold lower than that of the WT.^90^ In a case‐control investigation conducted in England, the United Kingdom, the vaccine efficacy (VE) against symptomatic disease caused by the Omicron or Delta variants was evaluated. The results showed the efficacy of ChAdOx1 15 weeks following two doses was 41.8% against the Delta variant. However, the efficacy against the Omicron variant was negligible.^91^ The efficacy of BNT162b2 was also impaired against the Omicron variant. The efficacy against the Delta variant was 63.5%, and only 34%–37% against the Omicron variant.^91^ In another case‐control study, the efficacy of mRNA‐1273 against the Omicron and Delta variants was assessed. According to the results, at 14–90 days after standard two doses of mRNA‐1273, the efficacy against the Omicron and Delta variants were 30.4% and 62.5%, respectively.^92^ The vaccine effectiveness data of the ChAdOx1, BNT162b2, and mRNA‐1273 vaccines against WT SARS‐CoV‐2, Delta, and Omicron variants is summarized in Table 1.^91^, ^93^, ^94^, ^95^, ^96^, ^97^, ^98^, ^99^, ^100^

**TABLE 1:** VEs of three different vaccines against severe acute respiratory syndrome coronavirus 2 (SARS‐CoV‐2) wild‐type (WT) virus, Delta, and Omicron variants

**Vaccines**	**WT (VE)**	**Delta (VE)**	**Omicron (VE)**	**Reference**
ChAdOx1	Lower at ∼70%	60% (95% CI, 53% to 66%); 41.8% (95% CI, 39.4% to 44.1%)	Negligible	^91^, ^93^, ^94^
BNT162b2	94.8% (95% CI, 89.8% to 97.6%); 95% (95% CI, 91% to 97%)	63.5% (95% CI, 61.4% to 65.5%); 79% (95% CI, 75% to 82%)	34%–37%	^91^, ^93^, ^95^, ^96^, ^97^
		88%		
mRNA‐1273	94.1% (95% CI, 89.3% to 96.8%)	86.7% (95% CI, 84.3% to 88.7%)	23.5% (95% CI, 16.4% to 30.0%)	^92^, ^98^, ^99^
		68.9% (95% CI, 60.1% to 75.8%)		

Increasing antibody levels in the body, for example, through taking booster doses of vaccines, can significantly increase the protection against SARS‐CoV‐2 infection, including against the Omicron variant. Indeed, the vaccine efficacy after a booster dose of vaccine was 60%–72% against the Omicron variant. However, this is still lower than that against the Delta variant (∼90%).^101^, ^102^ Such discrepancies in vaccine efficacy against the Delta and Omicron variants were also found in a phase IV clinical trial investigating the efficacy of booster doses of the mRNA vaccine and viral vector vaccine.^103^

### 3.4 Antibody evasion by Omicron

mAb therapy has shown considerable efficacy in the treatment of COVID‐19.^104^ The administration of mAbs can effectively reduce the viral load, death, and hospitalization.^105^ Most of the licensed or under development anti‐COVID‐19 mAbs target the spike protein to inhibit the interaction between SARS‐CoV‐2 and ACE2. Among them, the majority of mAbs target the RBD, with a few of them targeting the N‐terminal domain (NTD). However, as discussed above, mutations in the spike protein could compromise the binding affinity of mAbs to the spike protein, leading to a reduced treatment efficacy.^106^ Therefore, the large number of mutations in the spike protein of the Omicron variant may impact the efficacy of most mAbs developed for WT SARS‐CoV‐2 or other variants.

A high‐throughput yeast surface display screening identified 247 human neutralizing antibodies, which were clustered into six epitope groups. The Omicron variant spike protein mutations G446S, E484A, Q493A, and K417N conferred protection against neutralizing antibodies in four of these six epitope groups. Antibodies in the other two epitope groups display broad neutralizing activity against sarbecovirus in general. Interestingly, the Omicron variant is sensitive to the neutralizing activities of antibodies in these two epitope groups. Nevertheless, it is a concern that overall, the Omicron variant shows resistance to more than 85% of the 247 examined neutralizing antibodies.^107^ A number of mAbs are licensed for therapeutic use (bamlanivimab, etesevimab, casirivimab, imdevimab regdanvimab, cigavimab, tiagevimab, sotrovimab, and adintreviman). With the exception of bamlanivimab, the other eight mAbs could effectively neutralize the Delta variant. However, little neutralizing potency against the Omicron variant of bamlanivimab, etesevimab, casirivimab, imdevimab, and regdanvimab was observed. In addition, the potency of those mAbs that showed efficacy against the Omicron variant (cigavimab, tiagevimab, sotrovimab, and adintrevima) was reduced by 2.8 to 453‐fold.^88^ In another study, the antiviral activity of 19 different mAbs against eight variants (Alpha, B.1.526, B.1.429, Delta, Gamma, Beta, Omicron, B.1.1.529 + R346K) of SARS‐CoV‐2 was evaluated by calculating the fold changes in IC50, compared with WT virus.^85^ Notably, the mAbs against SARS‐CoV‐2 could be generally divided into two groups: the RBD‐targeting mAbs and the NTD‐targeting mAbs. Moreover, based on the binding modes and the affinity to ACE2, the RBD‐targeting antibodies are further classified into four classes (Class 1–4). In this study, the Class 1 RBD mAbs include CB6, Brii‐196, 1–20, and 910–30; the Class 2 RBD mAbs include REGN10988, COV2‐2196, LY‐CoV555, 2–15; the Class 3 RBD mAbs include GEGN10987, COV2‐2130, S309, 2–7, Brii‐198; the Class 4 RBD mAbs include ADG‐2, DH1047, 10–40, S2 × 259; the NTD mAbs include 4–18 and 5–7. The results showed that the IC50 of Class 1 and Class 2 mAbs for Omicron changed by −100 to −1000‐fold, compared to WT virus, which represents the sharpest decrease in all investigated variants in this study. The change of IC50 of Class 3 and Class 4 mAbs for Omicron were relatively mild than those of Class 1 and Class 2 but were still significantly lower than those for other variants. As for the two tested NTD mAbs, the IC50 for Omicron manifested −125 and −30‐fold change, compared to WT SARS‐Cov‐2.^85^ The mAb evasion level by five VOCs is summarized in Table 2.

**TABLE 2:** The resistances to variants of concern by different monoclonal antibodies

	**RBD mAbs**																	**NTD mAbs**	
**Fold change in IC50, compared with WT**	**CB6**	**Brii‐196**	**1‐20**	**910‐30**	**REGN10933**	**COV2‐2196**	**LY‐CoV555**	**2‐15**	**REGN10987**	**COV2‐2130**	**S309**	**2‐7**	**Bril‐198**	**ADG‐2**	**DH1047**	**10‐40**	**S2 × 259**	**4‐18**	**5‐7**
B.1.1.7 (Alpha)	−8.8	2.6	−5.2	−15	1.6	1.8	1.6	2.2	2.9	1.7	1.1	2.3	4.1	1.7	2.2	1.4	1.4	−5.1	−4
B.1.351 (Beta)	−196	2	−40	−60	−78	−2.5	−590	−1329	1.5	1.5	1.2	1.9	−1.5	1	3	−2.9	1.2	−39	−8.4
P.1 (Gamma)	−196	2.2	−16	−60	−121	−2	−590	−1329	1.9	1.1	1.1	1.2	1.8	−1	3	−2.2	1.2	−39	−74
B.1.617.2 (Delta)	2.1	1.2	−1.1	2.5	1.2	1.4	−590	−10	−1.8	−1.7	1.2	−1.1	−8.9	1	1.4	−1.8	−1.4	−39	−74
B.1.1.529 (Omicron)	<−1000	−134	<−388	<−159	<−1000	−140	<−1000	<−1000	<−1000	−390	−2.5	−231	2.2	−43	−124	−11	−35	−125	−30
Reference	^85^																		

### 3.5 Decreased neutralization of convalescent sera against Omicron

Individuals exposed to SARS‐CoV‐2 produce antibodies, which display neutralization activity. However, a study collected sera from recovered patients infected with WT SARS‐CoV‐2. Serum samples were used to determine the ability to neutralize pseudotyped Omicron and other VOCs and VOIs.^108^ The results revealed an 8.4‐fold decrease in the mean neutralizing activity against Omicron, compared to the D614G reference strain. In contrast, the neutralizing activity of other VOC and VOI pseudotypes was only about 1.2–4.5‐fold lower. Comparing the neutralization activity of 10 convalescent sera against WT SARS‐CoV‐2 and the Omicron variant showed a 32‐fold higher neutralization activity against the WT SARS‐CoV‐2.^85^ This is consistent with another pseudovirus neutralization study of the neutralizing activity of 180 convalescent serum samples previously infected with WT SARS‐CoV‐2.^37^

In comparative studies investigating the neutralizing activity of convalescent sera against all of the major SARS‐CoV‐2 variants (Alpha, Beta, Gamma, Delta, Lambda, Mu, and Omicron), a substantial reduction in the neutralizing titer was found against the Omicron variant (reduced by eight to 10 folds) versus other variants.^107^, ^109^ This reduction in convalescent sera efficacy against the Omicron variant may result in reinfections. A South African study, at the outset of the Omicron outbreak, demonstrates that the effective spread of the Omicron variant is related to an increase in the frequency of in‐transmission infections.^110^ Another study assessed whether prior infection with SARS‐CoV‐2 had a protective effect against reinfection with other variants. Results showed that prior infection provided more than 90% protection against reinfection of Alpha, Beta, and Delta variants, but this protection against reinfection was reduced by more than 30% for the Omicron variant.^111^

**FIGURE 3:**
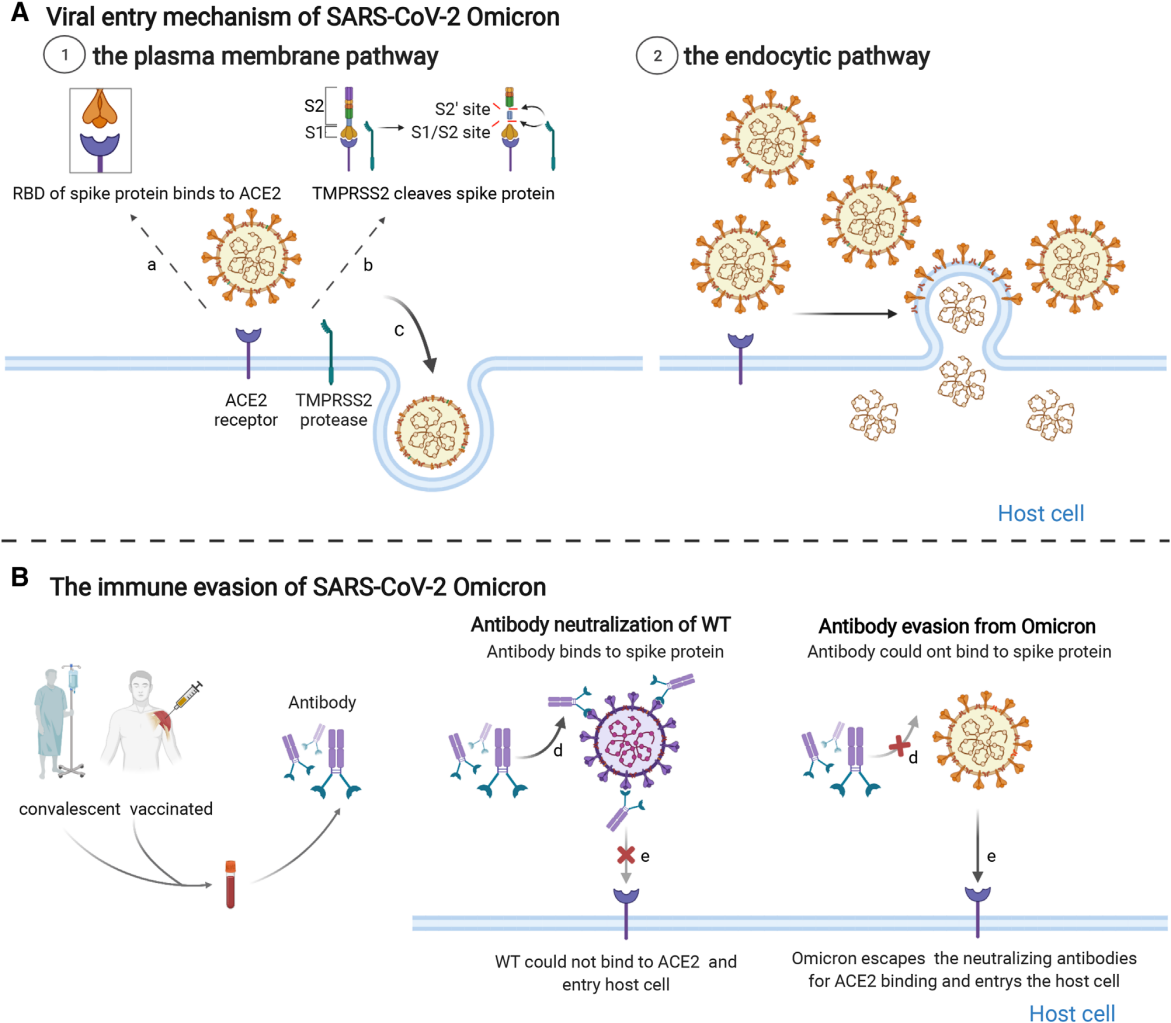
The impaired inhibition of neutralizing antibodies against Omicron variant infection. (A) Two cell entry pathways of SARS‐CoV‐2 Omicron. Left is the plasma membrane pathway using transmembrane protease serine type 2 and right is the endocytic pathway. (B) The immune evasion of Omicron from neutralizing antibodies. (1) Neutralizing antibodies from individuals prevent the wild‐type (WT) SARS‐CoV‐2 viruses into host cells by neutralizing viruses. The Omicron variant largely escaped neutralization by convalescent and vaccinated sera; therefore, viruses successfully entered into host cells by binding to the angiotensin‐converting enzyme 2 receptor

So far, a large (and growing) body of evidence suggests that the Omicron variant displays increased resistance to neutralizing antibodies induced either by vaccination or prior infection (Figure 3). However, the precise mechanisms through which a large number of mutations in the Omicron variant facilitates immune escape from the actions of neutralizing antibodies remains to be established.

## 4 STRATEGIES AGAINST THE OMICRON VARIANT

### 4.1 Developing effective vaccines against the Omicron variant

The importance of a vaccine against COVID‐19 was recognized very early in the pandemic, and vaccine development proceeded at an incredible speed. At the time of writing this review, there are 147 vaccines in the clinical trial, with another 195 in pre‐clinical development.^112^ Of the various licensed vaccines, over 90 billion doses have been administered (data from WHO). These vaccines have dramatically altered the trajectory of the COVID‐19 pandemic, particularly at preventing severe disease and reducing mortality.^113^

However, the large number of mutations found in the Omicron variant has led to a substantially higher infection rate than other VOCs.^91^, ^114^, ^115^, ^116^ After full vaccination of different types of COVID‐19 vaccines, the neutralizing antibody titer against the Omicron variant was low, indicating a below par protection against the Omicron variant.^116^ Previous studies have revealed that vaccines targeting mutated SARS‐CoV‐2 spike proteins elicited higher levels of neutralizing antibodies against variants than against WT SARS‐CoV‐2. This raises the possibility, and the significance, of exploring variant‐specific vaccines, particularly against the Omicron variant.^117^

At present, some mRNA vaccines containing partial mutations of the Omicron or Delta variant spike protein have been reported, based on the RBD of WT, Delta, Omicron, and hybrid (incorporating mutations of Delta and Omicron).^118^ The Omicron‐specific vaccine elicited the highest neutralizing antibodies against the Omicron variant. Interestingly, both the Delta and hybrid vaccines were also able to elicit broad immune protection in all VOCs, including the Omicron variant. However, the Omicron‐specific, as well as the hybrid mRNA vaccines, provide only limited protection against other variants.^118^ New RNA‐based vaccine technologies are also being developed. Circular RNAs (circRNAs) are covalently closed single‐stranded RNA transcripts produced by back‐splicing the pre‐mRNA of exons.^119^, ^120^ One such circRNA encoded the trimeric RBD of the Omicron spike protein.^121^ This vaccine candidate elicited high levels of neutralization antibody titers against the Omicron variant. However, it induced little neutralizing antibodies against other variants, while circRNA^RBD‐Delta^ vaccine could induce an intense antibody titer against Delta and Omicron. The development of Omicron‐specific vaccines remains a promising area of research and a viable strategy to counter the threat posed by the Omicron variant.

Contrary to the above results, a study reported that the levels of neutralization antibodies induced by an Omicron‐specific booster dose were lower, indicating that an Omicron‐specific vaccine may not provide sufficient immunity or protection, compared to the current WT spike‐based vaccines.^122^ Another study used techniques such as geometric deep‐learning to predict the relationship between mutations in Omicron and its antigenicity.^121^ It showed that those mutations decreased the antigenicity of Omicron in general. The results of in vivo experiments also validated this conclusion: After immunizing mice with recombinant RBDs proteins of WT and other five VOCs variants, antibody potency of sera from the Omicron group was much lower than that of any other variants.^121^

It is therefore unclear at the moment whether an Omicron‐specific mRNA vaccine will confer significantly stronger immunity in the population against the Omicron variant. More pre‐clinical and clinical data are needed to confirm this preliminary result. However, the vaccine developed for Delta was also found to be effective against Omicron in the above studies. Hence, the Delta‐specific vaccine may be an effective vaccine against Omicron.

### 4.2 Getting boosters of COVID‐19 vaccine

The development and clinical trials of effective vaccines against Omicron will take some time before it becomes generally available. It is difficult to envisage the future trajectory of the COVID‐19 pandemic, where the emergence of additional variants is possible. Therefore, existing vaccines remain the single most important and effective intervention to mitigate the effects of COVID‐19 infection. Since the effectiveness of the current COVID‐19 vaccines is expected to wane over time, booster doses of vaccines are necessary to maximize protection against SARS‐CoV‐2 infection.

The standard two‐dose regimen of COVID‐19 vaccination showed limited protective effects against the Omicron variant.^123^ The protective effect, neutralizing activity, against the Omicron variant is lost in 50% of individuals within 3 months after two doses of the mRNA‐1273 and BNT162b2 vaccines.^124^ However, a significant increase in neutralizing antibody levels was observed after a booster dose.^125^, ^126^, ^127^ After a single dose of BNT162b2 booster, neutralizing activity could be detected among all participants and increased sharply (by > 100‐fold). Notably, it was even higher than that against WT after two doses of BNT162b2.^128^ This is translated to a higher vaccine efficacy. In addition, according to the pseudovirus assay, the three‐dose vaccine was 10 times more effective in inhibiting virus viability than that of the two‐shot vaccine.^129^ In a study involving 16,087 Omicron‐positive cases, a booster dose of mRNA COVID‐19 vaccines had an efficacy against symptomatic disease of 61% (vs. 36% without the booster dose).^130^ While this is still very far from the original vaccine efficacy reported (over 90%), the above results nevertheless highlighted the importance of getting booster doses of the COVID‐19 vaccine.

**FIGURE 4:**
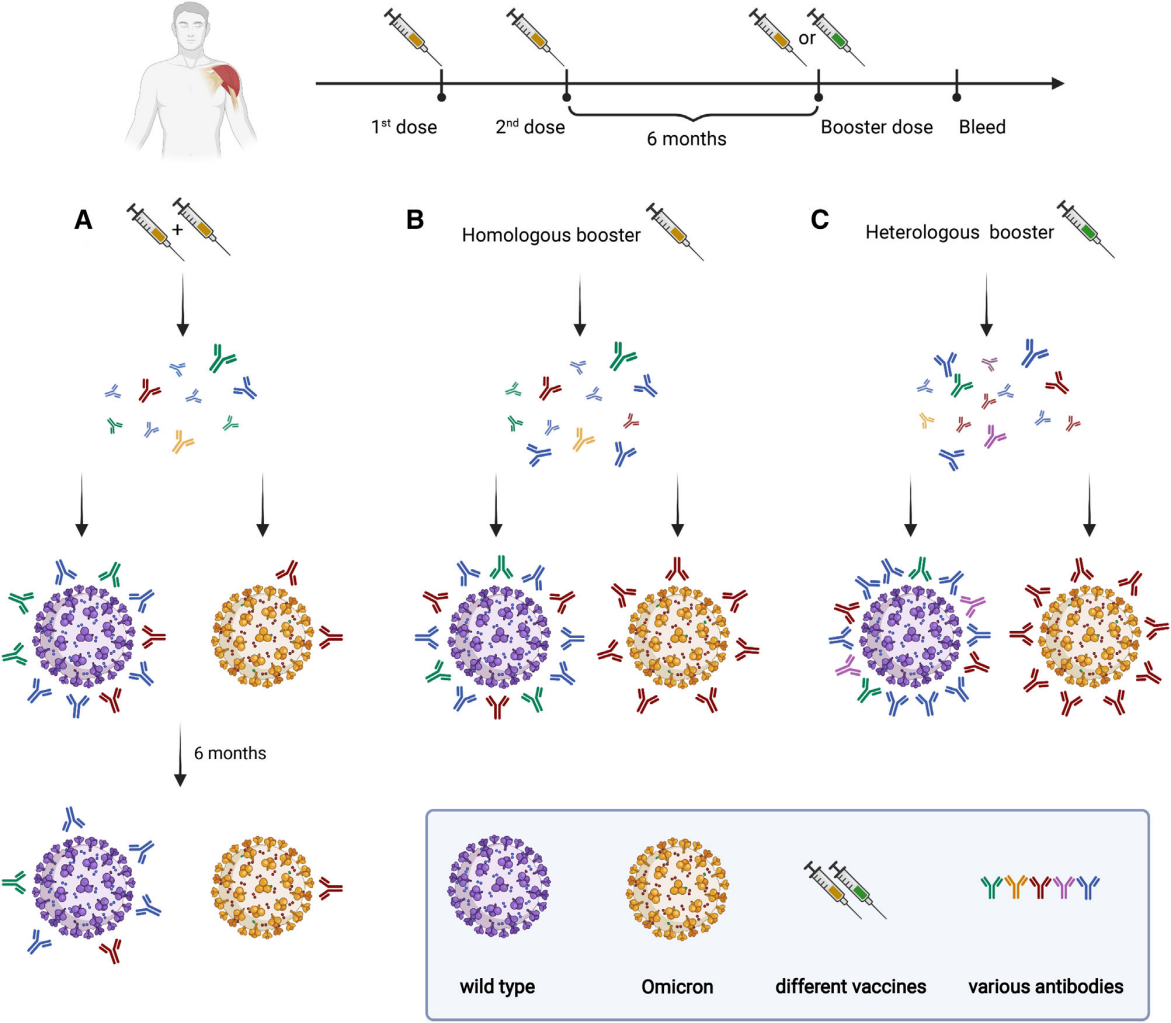
The protective effects of Coronavirus disease 2019 (COVID‐19) booster vaccines. (A) Antibodies produced by two doses of COVID‐19 vaccine showed effective protection against WT SARS‐CoV‐2 virus but reduced protection against Omicron variant between 14 days to 6 months after the second dose of vaccine. After the full vaccination, the protective effects decreased against both WT SARS‐CoV‐2 virus and Omicron variant. (B), (C) the heterologous booster (C) evaluated higher protective effects against both WT SARS‐CoV‐2 virus and Omicron variant than the homologous booster (B)

As described above, a number of vaccination strategies and technologies are currently available. With only a few exceptions, the majority of the first two doses of COVID‐19 vaccination are delivered using a homologous approach.^131^ However, the importance of getting booster doses, irrespective of a third or even fourth or fifth dose, raises the possibility of applying heterologous booster doses. Initial findings suggest that a heterologous booster vaccination strategy offers superior immunogenicity to a homologous approach.^131^, ^132^, ^133^ This may be due to an increased breadth of immune response in the case of heterologous vaccination strategies. A study gave four different boosters (30/15 μg BNT162b2, ChAdOx1, BBIBP‐CorV) for the participants who had been vaccinated with two doses of CoronaVac or ChAdOx1. The result revealed that heterologous boosters could induce the production of large amounts of antibodies against Delta and Omicron, compared with homologous boosters.^134^ Therefore, obtaining heterologous boosters might be an effective strategy to increase the intensity and persistence of the immune response (Figure 4).

### 4.3 Improving vaccine coverage

The initial large‐scale outbreak of the Omicron variant in South Africa corresponds to the relatively low vaccination rate (27.67%, data from WHO). This is substantially lower than many countries worldwide, such as the United States (62.0%), France (74.4%), and China (83.3%). It is conceivable that the weak immune barrier in South Africa provided an opportunity for the spread of the Omicron variant.^45^ However, given the large number of mutations in the Omicron variant and the reduced vaccine efficacy against Omicron infection, it is possible that the Omicron variant is capable of widespread infection even in regions with a high vaccination rate.

While vaccination reduces the chances of COVID‐19 infection, it dramatically reduces the chances of developing severe disease and/or mortality. These factors have important consequences, particularly for local healthcare systems. When the Delta variant was the predominant strain in the United States, hospitalization rates were 12.9 times higher in those who were not vaccinated. Similarly, hospitalization rates were 5.3 times higher in the unvaccinated group when the Omicron variant was dominant.^135^ These are reflected in clinical studies where two doses of the BNT162b2 vaccine have an efficacy against hospital admission of 70% and 93% for the Omicron and Delta variants, respectively.^136^ Similarly, another research from the UK illustrated that without being vaccinated before Omicron infection, the hospitalization rate was 0.76. But after two doses of AstraZeneca or Pfizer vaccine, that number would drop to 0.37 and 0.26.^137^ This highlights once again the importance of vaccination and getting booster doses.

### 4.4 Containing the spread of the Omicron variant

Effective and widespread vaccination remains one of the most important strategies to prevent the spread of SARS‐CoV‐2 and its variants. However, in the absence of a highly effective vaccine, which offers long‐lasting protection and also effective treatment options, non‐pharmacological interventions are vital to reduce transmission of SARS‐CoV‐2. These interventions, such as mask‐wearing, regular hand washing and social distancing are still currently recommended by the WHO. Efficient and quick testing, by polymerase chain reaction (PCR) or by rapid lateral flow antigen tests are also important approaches for the quick identification of infected individuals. Due to the unique H69/V70 deletion in the spike gene, the analysis of Omicron sequences would show *S*‐gene target failure (SGTF), which helps to diagnose Omicron accurately. However, compared to BA.1, sublineage BA.2 lacks such deletion, resulting in not being detected by SGTF.^138^ Population‐level protection against SARS‐CoV‐2 (and their variants) can also be monitored by regular antibody testing, either through traditional enzyme‐linked immunosorbent assays or through rapid lateral flow‐based neutralizing antibody tests. Such monitoring, at least in the short term during large‐scale outbreaks, can guide decisions and policies regarding vaccination schedules.

## 5 CONCLUSION AND PERSPECTIVE

In conclusion, based on the current studies, the Omicron variant possesses the most mutations of all VOCs. These mutations are often associated with extraordinary ability to spread and immune evasion. The robust immune evasion capability allows Omicron to easily escape from existing mAbs and vaccines and increase the risk of reinfection, leading to a surge in infections. Therefore, Omicron poses a great threat to human public health and safety and impedes the restoration of ordinary life. Facing this challenge, measures such as increasing vaccine coverage, promoting boosters, especially heterologous boosters, keeping social distance, and wearing masks, could be taken to limit the spread of the Omicron variant and reduce the rates of infection, hospitalization, and death.

At the same time, many scientists are also working on developing Omicron‐specific vaccines, but their effectiveness remains to be supported by more data. Additionally, the broad neutralizing ability demonstrated by the Delta‐specific vaccine suggests the potential of developing Delta vaccines. In the future, understanding Omicron's immune evasion mechanism and host immune responses, mining other more conserved viral epitopes, and improving the immunogenicity of vaccines will all contribute to the design of better vaccines and antibodies against Omicron.

Institute for Health Metrics and Evaluation (IHME) forecast model suggests that the number of COVID‐19 infections may decline in March this year, but this does not mean the end of the SARS‐CoV‐2 pandemic.^139^ The SARS‐CoV‐2 can infect humans and animals on a continuous basis and produce new mutations in them. Therefore, the emergence of new variants is inevitable. Through the Omicron outbreak, we learned that appropriate countermeasures are necessary to delay or prevent the emergence of severe variants, such as increasing vaccination rates, conducting genomic surveillance and tracking efforts, and developing highly effective vaccines and antibodies based on structure or immune escape mechanisms. It is believed that more strategies will be available globally to fight against and overcome COVID‐19 in the future.

## CONFLICT OF INTEREST

Kang Zhang is an editorial board member of *MedComm*. Author Kang Zhang was not involved in the journal's review of, or decisions related to, this manuscript. The other authors have no conflicts of interest to declare.

## AUTHOR CONTRIBUTION

X.W. and K.Z. conceived the study and revised the article. D.A., T.L. and X.H. wrote the paper. D.A. made the figures. J.L. and L.C. made the tables. D.T.B.‐H. revised the article.

## ETHICS STATEMENT

Not applicable.

## Data Availability

The data included in this study are available upon request from the corresponding author.
